# Lignin-derived carbon material for electrochemical energy storage applications: Insight into the process-structure-properties-performance correlations

**DOI:** 10.3389/fbioe.2023.1121027

**Published:** 2023-03-17

**Authors:** Wenqi Li, Jian Shi

**Affiliations:** Biosystems and Agricultural Engineering, University of Kentucky, Lexington, KY, United States

**Keywords:** Lignin, biorefinery, energy storage material, supercapacitor, lithium-ion battery, process-structure-properties-performance relation, thermochemical conversion, carbonization

## Abstract

As increasing attention has been paid to applications of lignin-derived energy storage materials in the last decade, most studies pursue the improvement of electrochemical performance obtained from novel lignin sources, or structure and surface modifications of synthesized materials, while the study on the mechanisms of lignin thermochemical conversion is rare. This review emphasizes on establishing a process-structure-properties-performance correlation across multiple key aspects associated with valorizing lignin from a byproduct of biorefineries to high performance energy storage materials. Such information is the key to a rationally designed process for the low-cost production of carbon materials from lignin.

## 1 Introduction

Energy is the driving force of global economic development and prosperity. However, there are increasing concerns about the environmental and societal problems especially the alarming greenhouse gas emissions caused by burning fossil fuels, which account for over 80% of today’s energy consumption (Hosenuzzaman et al., 2015; Masson-Delmotte et al., 2018). The biorefinery has the potential to replace a large fraction of fossil resources in plant-based feedstocks to satisfy energy demands, as well as to produce value-added building block chemicals and materials (Cherubini, 2010). However, the production cost of lignocellulosic biofuels is still far from competing with that of fossil fuels, despite various attempts to improve the viability of lignocellulosic biofuels by increasing enzyme efficiency (Hatakka, 1994), more efficient pretreatment technologies, (Socha et al., 2014; Li et al., 2018a), and genetically modified biomass feedstocks (Liu et al., 2020). Recently, biofuels community are paying increasingly attention on depolymerizing and upgrading lignin as building blocks for value added chemicals and materials, since lignin is one of the three primary compositions along with cellulose and hemicellulose, but the value of lignin has been underestimated.

Lignin is the second most abundant polymer on Earth (Thakur et al., 2014). As projected by the 2009 Renewable Fuel Standard (RFS2) on the basis of the Energy Independence and Security Act of 2007, a total of 36 billion gallons of renewable fuel is required, along with 15 billion tons of conventional biofuels (Langholtz et al., 2016). At the same time, approximately 60 million dry tons of lignin annually will be generated as a byproduct from the lignocellulosic biorefineries in the US alone by year 2022 (Ragauskas et al., 2014). This will add to the existing ∼100 million tons of lignin from the paper and pulping industry (Chakar and Ragauskas, 2004). Despite its great potential as a feedstock for various high-value chemicals, valorization of lignin is still challenging due to the complex structure and compositional heterogeneity of lignin (Ragauskas et al., 2014). Therefore, other potential applications of lignin are being explored to improve lignocellulosic biorefineries’ economic viability (Ragauskas et al., 2014).

With the fast development of consumer electronics and electric vehicles in the recent decades, the requirement for high performance energy storage materials boosts significantly. The global lithium-ion battery market is projected to grow from $ 41.1 billion to 116.6 billion with an annual growth rate of 12.3% from 2021 to 2030 (Markets, 2021); while the global Electric Double-layered Capacitors (EDLC) market is expected to grow from $ 3.27 billion in 2019 to $ 16.95 million at an annual growth rate of 23.3% from 2020 to 2027 (Research A.M, 2020). To meet the ever-increasing demand for the energy storage materials, tremendous efforts have been devoted to the research and development of the materials with low costs while high-power and high-energy density. As an abundant, low-cost, bio-renewable while long-term underutilized carbon source, lignin-derived carbon materials have attracted an increasing attention as energy storage applications, primarily including electrode material for supercapacitor (Li et al., 2018b), lithium-ion (Li et al., 2020a) and sodium-ion batteries (Li Y et al., 2016).

Despite the increasing interests in lignin-derived carbon materials for electrochemical energy storage purposes, the potential applications and electrochemical performance of those synthesized carbon materials have been of more interest, and such progresses have been well summarized by multiple review articles (Liu et al., 2015; Kai et al., 2016; Gao et al., 2017; Wu et al., 2020); While limited literatures are available on linking the characteristics of biorefinery derived lignin, the chemistry of thermochemical conversion processes, the chemical structure and the properties of the lignin-derived carbon materials, together with the performance of the synthesized electrode. Such information is the key to a rationally designed process for the low-cost production of carbon materials from lignin.

Herein, we provide a critical review that emphasizes on the process-structure-properties-performance correlation across multiple key aspects associated with valorize lignin from a byproduct of biorefineries to high performance energy storage materials. The reaction mechanisms as well as the effects of distinct thermochemical conversion processes on carbonization process of lignin are covered in Section 2. Instead of simply reporting a plenty of impressive electrochemical test results obtained from the diverse lignin-derived energy storage materials, Section 3 discusses a variety of recently developed strategies and related mechanisms associated with synthesizing energy storage materials from lignin with high performance. Following up, recent research advances in analytical methodologies towards better understand the process-structure-property-performance correlation were summarized in Section 4. Finally, future research directions and opportunities related to improving properties of the lignin derived functional materials and commercialization are outlined in Section 5. Collectively, we hope to extract and crystalize the critical elements and insights on the process-structure-properties-performance correlations toward rational design of high performance and low-cost lignin derived carbon material for electrochemical energy storage applications.

## 2 Carbonization through thermochemical conversion processes

Carbon has numerous allotropes, such as diamond, graphite, graphene, nanotube, fullerene etc., because of its valency. With good electric conductivity and tailorable structure and surface (shape, surface area, porosity, and pore size distribution), carbon materials have demonstrated potentials in diverse functional material applications, such as electrochemical energy storage, absorbent, catalyst, soil amendment, etc. Recently, the growing demands in carbonaceous functional materials, as well as urgent need to replace fossil fuels with sustainable alternatives lead to increasing interests to synthesize low-cost carbon materials from biomass with desired functions. Pyrolysis and hydrothermal carbonization, as primary thermochemical conversion processes, have been widely applied to the synthesis of biomass-derived carbon materials. Additionally, other pyrolysis technologies, such as laser pyrolysis and plasma pyrolysis were also reported to be applied to synthesize lignin-derived carbon materials. The choice of thermochemical conversion technology, as well as process conditions deliver totally different reaction mechanisms which significantly influence properties of synthesized carbon materials.

### 2.1 Pyrolysis carbonization of lignin

Pyrolysis is a process for thermal decomposition of materials at elevated temperatures in an inert atmosphere, which has been intensively studied in recent years (Patwardhan, 2010; Li et al., 2017; Li W et al., 2020). Based on the heating rates, pyrolysis can be categorized into fast pyrolysis and slow pyrolysis. Fast pyrolysis, carried out at enhanced temperatures (around 500°C), high heating rate (up to 2000°C/s) and short reaction times (<2 s), has been demonstrated as an effective method for bio-oil production from biomass (normally around 60 to 70 wt%) in addition to a small amount of biochar (20%) and gases (15%); while slow pyrolysis, remaining a slow heating rate and long reaction times (up to days), primarily converts biomass to high yield of biochar (around 50%) at the expense of the volatile products (Li W et al., 2020).

The behavior of lignin in the pyrolysis process is affected by several factors, including the lignin origin (i.e., softwood, hardwood and grass lignin) (Li et al., 2018b), lignin extraction/pretreatment method (Zhou et al., 2016), pyrolysis heating rate and reaction temperature (Ferdous et al., 2002), and selection of catalyst (Yang et al., 2007). The main products acquired from the lignin pyrolysis includes gaseous compounds (e.g., H_2_, CH_4_, CO, and CO_2_), phenolic monomers and other poly-substituted phenols. Besides the abovementioned liquid and gaseous compounds, depending on pyrolytic temperature and heating rate, a fraction of lignin is converted to a thermally stable solid product, usually referred to as biochar. With plenty of surface functional groups (e.g., C-O, C=O and OH), these carbon materials have potentials to be applied to functional materials when subjected to various functionalization processes.

Despite that, there is an agreement that lignin pyrolysis involves with lignin depolymerization and phenoxyl free radicals chain reactions (Hu et al., 2012), the formation mechanism of monomeric and oligomeric products evolved during pyrolysis has not been fully elucidated (Demirbaş, 2000; Murwanashyaka et al., 2001; Branca et al., 2003; Ben and Ragauskas, 2011; Zhou, 2013; Bai et al., 2014; Li et al., 2017). Some researchers believe that lignin pyrolysis will first be cracked into numerous phenolic oligomers. Those oligomers will be further depolymerized into a variety of phenolic monomers during a series of secondary reactions (Piskorz et al., 1999). Afterwards, a competing model suggested that the oligomers were formed from secondary reactions through repolymerization of monomers derived from the primary reactions (Patwardhan et al., 2011).

Compared to cellulose and hemicellulose pyrolysis, the reaction mechanism of pyrolysis carbonization of lignin is more complex due to the heterogeneity in lignin composition. It is believed that lignin pyrolysis is involved with multiple reaction phases, including prime and secondary reactions (Liu et al., 2015; Li et al., 2017). The prime reactions of lignin pyrolysis are related with the cleavage of β-*O*-4 linkages to generate vinylphenols (Li et al., 2017; Li W et al., 2020). The primary products undergo a series of secondary reactions to produce a variety of H, G and S type monomers. There is a general agreement that lignin pyrolysis involves with free radical chain reactions (Kim KH et al., 2015) and the monomer products are presented as free radicals (Li et al., 2017; Li et al., 2020b). Since free radical reaction are chain reactions, it would not terminate as long as the free radicals are present. Hence, the originally volatilized H, G and S type would subsequently undergo repolymerization and condensation into oligomers and finally form solid fractions, namely char and coke (Li W et al., 2020). With the heating rate reduced in slow pyrolysis, the free radicals are more likely to be repolymerized to form polycyclic aromatic hydrocarbons (PAHs) and coke (Li W et al., 2020).

### 2.2 Hydrothermal carbonization (HTC) of lignin

Hydrothermal carbonization is a wet biomass thermochemical conversion technology, which mimics the natural process of coal formation but in a much shorter period of time. Being placed in a closed reactor, such as an autoclave, the biomass or biomass-based precursors are surrounded by water and treated at approximately 130°C–280°C under self-generated steam pressure. The final products include solid residue, referred to as hydrothermal biochar, soluble organic compounds and gaseous products, mainly composed of CO_2_. Compared to pyrolysis carbonization, HTC generates more biochar while less gases (Sugimoto and Miki, 1997). In addition, with higher H/C and O/C ratios, the chemical structure of hydrothermal biochar is more analogous to natural coal rather than pyrolytic biochar (Van Krevelen, 1993). The primary advantage of HTC over pyrolysis is that it can directly deal with the high-moisture biomass feedstock without energy-intensive preprocessing/drying (Pandey et al., 2015). However, the high pressure of HTC reaction requires special reactor and process design, leading to high capital investments. Additionally, continuous feeding solid biomass into reactors is challenging under high pressure, which hinders the scaling up of HTC process (Pandey et al., 2015).

Although it is believed that HTC process is generally governed by dehydration and decarboxylation (Hoekman et al., 2011), the complex reaction networks are not fully understood yet. So far, only a series of separate reaction mechanisms are proposed and identified, which include hydrolysis, dehydration, decarboxylation, polymerization and aromatization (Funke and Ziegler, 2010). Despite it is difficult for lignin to be completed hydrolyzed under the typical hydrothermal temperatures, partial hydrolytic reactions may start from the cleavage of the ester and ether bonds and end up into phenolics. Chemical dehydration significantly lowers the O/C and H/C ratios (Bergius, 1928). HTC causes partial elimination of carboxyl groups (Blazsó et al., 1986; Lau et al., 1987). When reaction temperature increases over 150°C, both carbonyl and carboxyl groups degrade rapidly and generate CO and CO_2_ (Murray and Evans, 1972). Depending on the severity of HTC condition, elimination of carbonyl and carboxyl groups during decarboxylation leaves plenty of unsaturated compounds that are very reactive and prone to repolymerization (Terres, 1952). The repolymerization contributes to formation of hydrothermal char on the surface of the non-hydrolyzed lignin or polyaromatic char. The rate of carbonization is determined by the degree of aromatic condensation. Alkaline condition appears to promote the aromatization (Nelson et al., 1984). It should be noted that all of the separate reactions mentioned above are not consecutive steps but rather a parallel pathway network, and that the extent of each reaction associated to the formation of the hydrothermal products primarily depends on the type of feedstocks and HTC conditions (Funke and Ziegler, 2010).

### 2.3 Laser abated direct carbonization of lignin

Laser pyrolysis is a novo technology developed for the synthesis of nanostructured materials, which involves complex photothermal and thermochemical processes. It attracts increasing attention, especially for the synthesis of carbide nanoparticles (e.g., SiC, ZrC, TiC) and ceramic nanoparticles (e.g., SiO2, TiO2) (Wang and Gao, 2019). During laser pyrolysis, a continuous wave CO2 laser is used to heat flowing reactant gases, resulting in lignin decomposition. It is estimated that a temperature of 4,000°C–10,000°C can be obtained within microseconds for a typical laser pyrolysis (Fanter et al., 1972). In addition to the ultra-fast temperature rise, the cooling rate of primary pyrolysis products generated by laser pyrolysis is reported as fast as its heating rate so that minimizer secondary reactions (Fanter et al., 1972).

**FIGURE 1:**
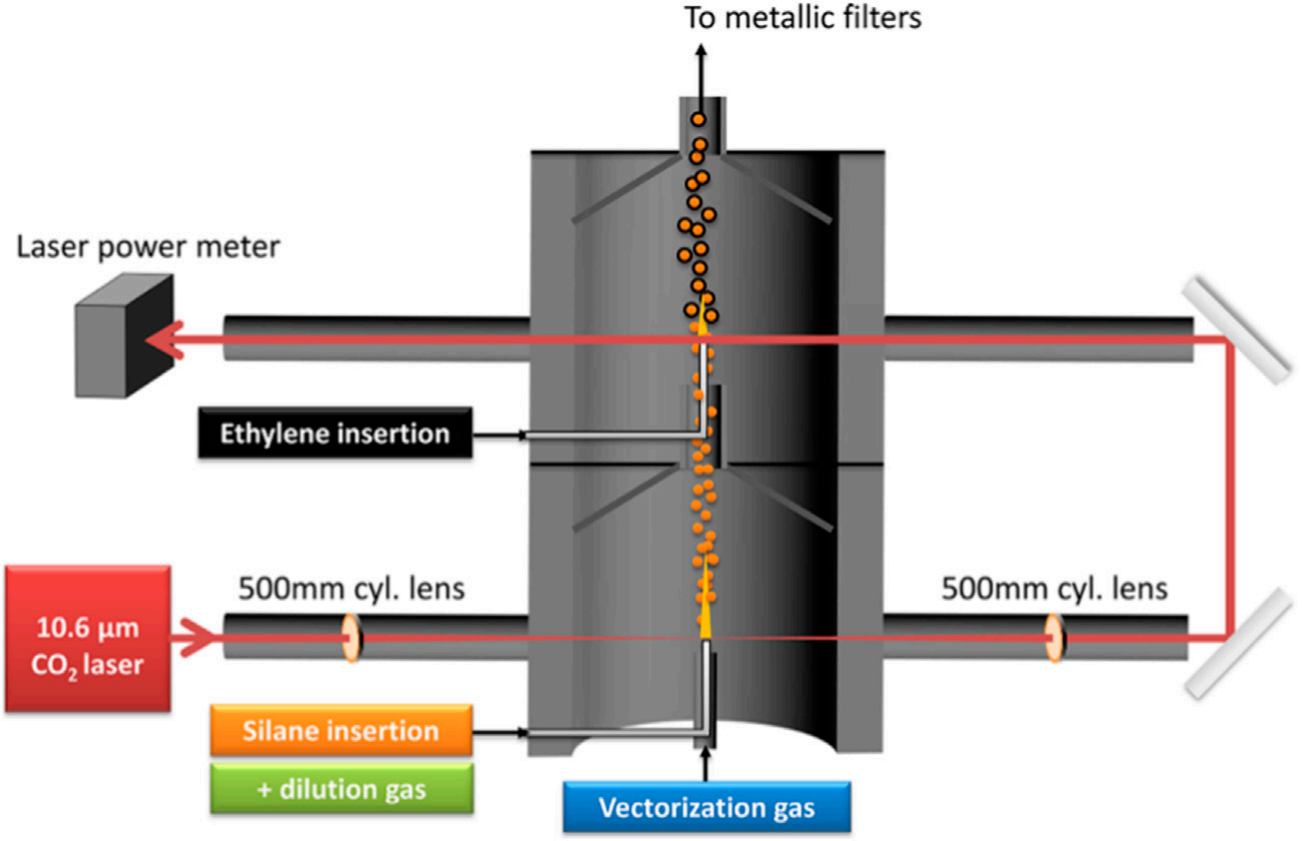
Schematic diagram of a laser pyrolysis reactor configuration (Sourice et al., 2015).

As a byproduct of bio-oil production, highly crystalline flake graphite was synthesized from mixture of Fe metal and biochar derived from a variety of biomass (sawdust, wood flour, corn cob, cellulose and lignin) through a laser pyrolysis process. The size of a “potato”-shaped flake graphite was in a range of 1–5 μm, which is determined by the physical dimensions of the metal particles. The flake graphite is reported essentially indistinguishable from high-grade commercial Li-ion grade graphite, though electrochemical tests were not been provided (Banek et al., 2018). In a separate study, laser pyrolysis was applied to synthesize a Si/C nanocomposite that used as negative electrode of lithium-ion batteries. Silane and ethylene were the precursors of Si and C, respectively. The merit of the laser pyrolysis in this study is its high efficiency since it allows precursor gases pass through the reactor and grow at the same time with a one-step synthesis process (Sourice et al., 2015), as shown in Figure 1.

However, laser pyrolysis highly depends on the optical properties of precursor materials (Mbonane, 2018). It might be necessary to introduce photosensitizers, such as sulfur hexafluoride, ethylene, silicon tetrafluoride, and ammonia, into carrier gas to initiate particle nucleation (Yefimov, 2009). Additionally, the confined area that laser beam focuses and limited layers that laser is able to transmit significantly restrict sample size, which propose challenges for large-scale production as well as difficulties to generate homogeneous electrode materials. Take the previous mentioned study as example (Banek et al., 2018), the diameter of laser beam was 2 mm each pellet had to be laser pyrolyzed individually and rotated at around 1.2 rpm. The laser exposed surface layer needed to be cut off from the unexposed core to obtain the result graphite flake. It is hard to obtain a preferred material in large scale and with a well-controlled quality *via* the method.

### 2.4 Thermal plasma pyrolysis

Plasma is ionized gas. When inert gas is presented in a strong electromagnetic field, free electrons in the inert gas will oscillate, which cause the free electrons to collide with atoms of the inert gas and remove electrons so that ionize the inert gas and form plasma. Thermal plasma generated by carbon electrodes can be dated back to 1960s. However, the short longevity of the electrodes was a big challenge that hindered wide application of the technology (Nema and Ganeshprasad, 2002). As the advances in more reliable and efficient torches for thermal plasma generation in the recent years, the use of thermal plasma as a heating source for pyrolysis and gasification has received increasing interests (Tang et al., 2013), especially for converting high calorific plastic waste into the syngas. The process of thermal plasma pyrolysis can be described as carbonaceous solid precursor reacts with limited amount of oxygen at the high temperature of highly reactive plasma region, where a plenty of electrons, ions and excited molecules generated under high energy radiation (Tang et al., 2013).

A microwave assist plasma carbonization process was developed to fabricate carbon fiber from polyacrylonitrile (Kim SY et al., 2015). When compared with conventional pyrolysis, plasma carbonized carbon fiber exhibited a higher surface roughness, which lead to an improvement of increase the mechanical properties of the CF Reinforced Polymer.

## 3 Advances in lignin-derived energy storage application

### 3.1 Supercapacitor

**FIGURE 2:**
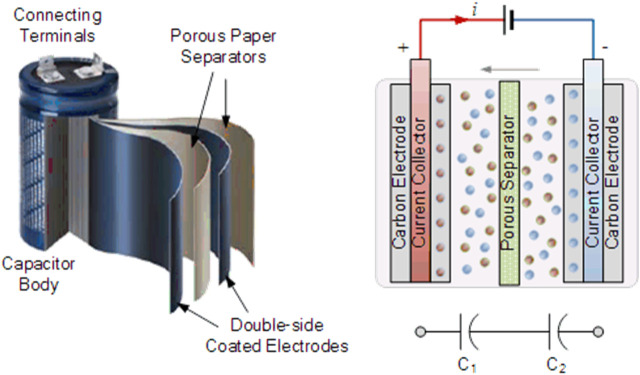
Configuration and working principle of an EDL supercapacitor (Tutorials, 2019)

A supercapacitor is a high-power energy storage device widely used in transportation vehicles, power grids and consumer electronics (Gu and Yushin, 2014). Compared to batteries, such as lithium-ion batteries (LIBs), supercapacitors are favored because of their high-power density and long lifespan, which are suitable for short-term energy storage and burst power delivery. As shown in Figure 2, a typical supercapacitor is made up of two conductive electrodes with high surface area, separated by an electron-insulating but ion permeable membrane and fully soaked in electrolyte. As the ions of electrolyte spontaneously transfer toward/apart the surface of electrodes with electrons moving between the two electrodes but no charge transfer occurring across the electrode and electrolyte interface during charging and discharging, the capacitance acquired is called electric double-layer (EDL) capacitance. The EDL capacitance is simply achieved by physical adsorption of ions without chemical reactions involved on the interface between electrolyte and electrodes during charging and discharging. Although EDL is mainly attributed to the primary capacitance originated from the electrode made of carbon materials, many carbon materials contain functionalities or have modification on their surface, which contribute to extra capacitance obtained *via* redox reactions between the electrolyte and electrode, referred as electrochemical pseudocapacitance (Winter and Brodd, 2004).

Based on the principles of EDL capacitance, porous materials, especially activated carbon, have received the most attention as electrode materials of supercapacitor not only due to their impressive surface area and electric conductivity but also their tailorable pore structure (shape, pore size distribution) (Zhai et al., 2011). Lignin has been considered as a favorable precursor for porous carbon materials owing to its high carbon content, highly branched and cross-linked structure, and low feedstock cost (Li et al., 2018b). Additionally, plenty of oxygen-containing functional groups formed on the surface of prepared carbon materials offer extra pseudocapacitance to the total capacitance (Saha et al., 2014). Therefore, lignin-derived carbon materials have received extensive investigation for the potential application as supercapacitor’s electrode in the last decade, with some literature summarized in Table 1. To obtain higher specific surface area and conductivity and tailored surface functionality for overall performance, various synthesis strategies have been examined in the lignin-derived carbon materials for supercapacitor application. Reviewed here are lignin-derived activate carbon and carbon fibers, surface modification of the lignin-derived carbon materials and lignin-derived carbonaceous composite materials.

**TABLE 1:** Summary of lignin-derived carbon materials for supercapacitor application.

Lignin source	Target materials	Carbonization	Activation	Modification	SSA (m^2^/g)	Capacitance (F/g)	Electrolyte	Ref.
Hardwood lignin	AC	Pyrolysis	KOH (700°C, 2 h)	N/A	907	165@ 50 mV/s	1 M H_2_SO_4_	C-Zhang et al. (2015)
Poplar extracted lignin;	AC	Pyrolysis (700°C, 1 h)	KOH (700°C, 1 h)	N/A	621.25;	86.7,	1 M H_2_SO_4_	Li et al. (2018b)
Pine extracted lignin					314.95	48.3 @ 0.5 A/g		
Alkaline lignin	AC	N/A	ZnCl_2_ (700°C, 1 h)	N/A	866,	142.09,	6 M KOH	Wu et al. (2017)
			KOH (700°C, 1 h)		1,191,	251.04,		
			K_2_CO_3_ (700°C, 1 h)		1,585	263.46 @ 50 mA/g		
Corn stover lignin	AC	Hydrothermal (180°C, 18 h)	KOH (800°C, 3 h)	N/A	1,660	420 @ 0.1 A/g	6 M KOH	Guo et al. (2017)
Black liquor lignin	AC	Pyrolysis (900°C, 2 h)	KOH (900°C, 2 h)	N/A	1,406	87 @ 5 mV/s	1.5 M NEt_4_BF_4_/ACN	Navarro-Suárez et al. (2014)
Alkali lignin	AC	Pyrolysis (500°C, 1 h)	KOH (800°C, 1 h)	N/A	3,775	286.7 @ 0.2 A/g	6 M KOH	Zhang et al. (2015b)
Kraft lignin, ethanol extracted lignin, alkali extracted lignin	AC	Pyrolysis (950°C, 6 h)	N/A	N/A	1,092,	91,	1 M H_2_SO_4_	Jeon et al. (2015)
					519,	35,		
					126	53 @ 0.5 A/g		
Softwood Kraft lignin	AC	N/A	KOH (800°C, 1 h)	N/A	1800	200 @ 10 A/g	EMIBF4	Klose et al. (2017)
Kraft lignin	AC	Pluronic F127	CO_2_ (875°C, 35 min)	N/A	624	102.3,	6 M KOH	Saha et al. (2014)
		Pyrolysis (1,000°C, 15 min)	KOH (1,000°C, 35 min)		1,148	91.7 @ 2 mV/s		
Acid washed lignin	AC	Pyrolysis (900°C, 15 min)	Pluronic P123, EO_20_PO_70_EO_20_	N/A	803	97.1 F/cm^-3^@ 289 mA/cm^-2^	6 M KOH	Li H et al. (2016)
Alcell lignin	AC	Pyrolysis (900°C, 2 h)	Zeolites Y template	N/A	1,085	250 @ 50 mA/g	1 M H_2_SO_4_	Ruiz-Rosas et al. (2014)
Alcell lignin	AC	Pyrolysis (900°C, 2 h)	Zeolites β template	N/A	930	140 @ 1 A/g	1 M H_2_SO_4_	Salinas-Torres et al. (2016)
Alkali lignin, low sulfur	ECNF mats	Pyrolysis (600°C, 1 h)	KOH (900°C, 2 h)	N/A	2005	205 @ 0.3 A/g	0.5 M Na_2_SO_4_	Ago et al. (2016)
Alkali lignin	ECNF mats	Pyrolysis (1,200, 1 h)	N/A	N/A	583	64 @ 0.4 A/g	6 M KOH	Lai et al. (2014)
Alkali lignin	ACF	N/A	KOH (850°C, 0.5 h)	N/A	N/A	344 @ 10 mV/s	6 M KOH	Hu et al. (2014)
Alkali lignin	ECNF mats	Pyrolysis (900, 2 h)	CO_2_ (850°C, 3 h)	N-doped	1,113	410 @ 1 A/g	6 M KOH	Yang et al. (2018)
Poplar lignin	AC	Hydrothermal (200°C, 24 h)	KOH (800°C, 1 h)	N-doped	2,218	312 @ 1 A/g	6 M KOH	Zhang et al. (2016)
Sodium lignosulfonate	AC	Pyrolysis (900°C, 3 h)	TEOS template	S-doped	1,054	328 @ 0.2 A/g	6 M KOH	Tian et al. (2017)
Kraft lignin	MnO_2_/ECNF mats	Pyrolysis (1,400°C, 0.5 h)	N/A	N/A	N/A	171.6 @ 5 mV/s	1 M Na_2_SO_4_	Youe et al. (2018)
Acetic acid lignin	Iron oxide/HCNF	Pyrolysis (900°C, 1 h)	N/A	N/A	281	121 @ 0.5 A/g	1 M Na_2_SO_3_	Yu et al. (2018)
Sodium lignosulfonate	NiO/AC	Pyrolysis (600°C, 2 h)	Pluronic F127	N/A	802	880.2 @ 1 A/g	6 M KOH	Chen et al. (2013)
Alkali lignin	Graphene/C	Hydrothermal (240°C, 16 h)	N/A	N/A	1804	190 @ 0.5 A/g	6 M KOH	Ye et al. (2017)
Sodium lignosulfonate	SWCNT/C	Hydrothermal (180°C, 12 h)	N/A	N/A	150.5	292 @ 0.5 A/g	1 M Li_2_SO_4_	Peng et al. (2018)

#### 3.1.1 Lignin derived activated carbon

The original biochar derived from thermochemical conversion has insufficient functional groups and limited surface area and porosity, which restrict the application of biochar as functional carbon materials. Abundant surface functional groups provide extra pseudocapacitance, while large surface area and appropriate pore structure facilitate efficient permeation of electrolyte. Therefore, activation is a critical upgrading step so that the original biochar is able to be used as a functional material for supercapacitor application.

According to International Union of Pure and Applied Chemistry (IUPAC), pores can be classified into macropores, mesopores and micropores (Zhai et al., 2011). A macroporous material is a material containing pores with diameters larger than 50 nm. Mesoporous materials contain pores with diameters between 2 nm and 50 nm while microporous materials have pores with diameters smaller than 2 nm. It is believed that a bimodal porous structure involving micropores and a narrow distribution of pores between mesopores and micropores is preferred of energy storage purpose (Zhai et al., 2011). The macropores are beneficial to the fast ion transportation while the mesopores and micropores offer sufficient surface area that bulk ion absorption relies on.

Therefore, to synthesize organized porous materials with narrow distributions of mesopores and micropores, a plenty of templates have been applied (Ruiz-Rosas et al., 2014; Saha et al., 2014; Li H et al., 2016; Salinas-Torres et al., 2016). In a typical templating process, a template agent such as Pluronic F127 (Saha et al., 2014) or colloidal silica (Fierro et al., 2013) is first infiltrated into the lignin precursor. The mixture is then subjected to pyrolysis to obtain the templated char. After the template agent is removed, the templated char is then subjected to either physical or chemical activation to enlarge pore volume and create connection between pores. Finally, activated carbons with tailored ratios of micro-, meso- and macropores are acquired. The advantage of templated activation is the tunable and organized pore structure. However, template agents are usually expensive and cannot be easily recycled, and highly toxic and corrosive acid, such HF or H_2_SO_4_ is required to remove the template agents. Both drawbacks impede its wide application.

In addition to template-directed approaches, other porous material synthesis technologies with activation agents are attracting increasing attention due to their advantages of template-free, easy operation, relatively low costs and environmental-friendly. Based on activation mechanisms, those activation processes can be divided into physical activations and chemical activations (Wigmans, 1989).

Physical activation with carbon dioxide, steam or a combination of them is a traditional manufactural process of activated carbon (Bansal et al., 1988). The reaction mechanisms involved with the CO_2_ and steam activation are shown as follows:

$$C+H_{2}O\;\to CO↑+H_{2}↑$$

$$C+{CO}_{2}\;\to 2CO↑$$

$$CO+H_{2}O\;\to {CO}_{2}↑+H_{2}↑$$

For physical activation, development of pore structures is following a combination of two mechanisms. 1) Pore drilling contribute to a steady increase in pore diameters; 2) Pore deepening that affect pore depth more than width (Wigmans, 1989). Activation of carbonaceous materials may happen only when initial pores are available. During physical activation, around 10%–20% of disordered carbon is first removed. The exposed carbon crystallites will be further burn off to develop pore volume. The pore volume develops either *via* drilling or deepening on the initial pores, the extent of the two mechanisms associate with pore development is determined by the type of feedstock and the process conditions (activation temperature, residence time, activation gas composition). The pore structure is highly dependent on molecular size and reactivity of the physical activation agents. Steam activation leads to more meso- and macropores, while carbon dioxide activation contributes to more micropores (Rodriguez-Reinoso et al., 1995). In comparison to steam activated carbon, carbon dioxide activated carbon delivered a wider range of pore size distribution.

Chemical activation is carbonization process by impregnate carbon precursor with certain chemicals, such as KOH, H_3_PO_4_, ZnCl_2_, etc. via pyrolysis at an enhanced temperature (700°C–1,000°C) (Liu et al., 2015). Chemical activation is preferred to physical activation due to better quality consistency, and shorter residence time required for activation.

KOH is one of the most commonly used chemical activation reagents. It is believed that the KOH activation reactions consist of several simultaneous and/or sequential reactions. KOH dehydrates at 400°C to produce K_2_O between KOH and carbon begin with solid-solid reactions, which are followed by gasification reactions (Liu et al., 2018). Carbon will be oxidized by water to produce CO, CO_2_ and H_2_. The reactions between K_2_O and CO_2_ intermediates finally produce K_2_CO_3_. The reaction processes can be illustrated as follows (Wang and Kaskel, 2012):

$$2KOH\;\to K_{2}O+H_{2}O$$

$$C+H_{2}O\;\to CO↑+H_{2}↑$$

$$CO+H_{2}O\;\to CO_{2}↑+H_{2}↑$$

$$CO_{2}+K_{2}O\;\to K_{2}CO_{3}$$

ZnCl_2_ has a high boiling point (732°C) while low melting point (290°C), which is beneficial to the diffusion of ZnCl_2_ throughout the carbon precursor matrix under low temperature while migrate throughout microporous network at high temperature. This characteristic contributes to an even and well-developed pore network (Gonzalez-Serrano et al., 1997). In despite of the merits, the activation process requires HCl to remove the ZnCl_2_ agent, which leads to extra costs on energy and pollutant treatment.

H_3_PO_4_ activation involves two steps: impregnation and thermochemical conversion. Impregnation allows the formation of a crosslink between phosphoric acid and lignin macromolecule and the biomass loading in H_3_PO_4_ significantly affect the properties of the activated carbon (Zuo et al., 2009a; Zuo et al., 2010). Lower biomass loading will remarkably enhance porosity of H_3_PO_4_ activated carbon. H_3_PO_4_ plays two roles in activation process. 1) promote bond cleavage, hydrolysis, dehydration, condensation and cross-linking reactions between H_3_PO_4_ and carbon precursor. 2) the H_3_PO_4_ may act as a template which promotes the formation of micropores (Zuo et al., 2009b).

In order to compares the effect of various activation agents on the lignin-derived porous carbon materials, Wu et al. synthesized activated carbons from alkaline lignin-by a one-step activation process using ZnCl_2_, KOH and K_2_CO_3_. Although the different chemical agent displayed little effect on the formation of functional groups at the surface of prepared carbon materials, it demonstrated a critical impact on the specific surface area (SSA). The SSAs of the ACs activated by ZnCl_2_, KOH and K_2_CO_3_ were 866, 1,191 and 1,585 m^2^/g, which lead to 142.09, 251.04 and 263.46 F/g, respectively, at a current density of 40 mA/g (Wu et al., 2017).

In addition to the different activation approaches, the effect of heterogeneity in structure and composition of lignin precursors on the structure of porous carbon material and supercapacitance performances were also investigated. Compared to pine (softwood) lignin-derived AC, poplar (hardwood) lignin-derived AC exhibited a higher level of specific surface area and volume of both mesopores and micropores because of the variations in structure and composition of lignin between softwood and hardwood. As a result, the poplar lignin-derived AC had a higher value of specific capacitance at each current scan rate than the softwood lignin-derived AC (Li et al., 2018b).

#### 3.1.2 Lignin-derived carbon fibers

In addition to AC, lignin-derived carbon fibers (LCFs) have also received increasing attention for supercapacitor application (Fang et al., 2017). A random packed LCFs mat is able to provide a relatively high surface area even without activation process (Lai et al., 2014). Additionally, it offers potential to develop flexible electrode materials due to its nature of mechanically flexible (Hu et al., 2014; Lai et al., 2014). Lai et al. prepared an electrospun carbon nanofiber (ECNF) mat through electrospinning aqueous mixture of alkali lignin and polyvinyl alcohol into fiber mat followed by stabilization and carbonization. As the mass ratio of lignin/PVA was 70/30, the ECNFs mat exhibited a specific surface area of 583 m^2^/g without the assistance of activation process. As a result, the ECNFs (70/30) mat delivered 50 F/g at a current density of 2 A/g and the capacitance retained 90% after 6,000 cycles. With the assistance of an activation process, the specific surface area of the ECNFs mat was enhanced significantly. Ago et al. produced ECNFs mat from lignin/PVA (75/25) as carbon precursor followed by a separated KOH process and obtained a superior specific surface area of 2005 m^2^/g, which led to a high value of specific capacitance of 205 F/g at current density of 0.3 A/g.

##### 3.1.2.1 Surface modification of lignin-derived carbon materials

Pseudo-capacitance is the electrochemical storage of electricity in an electrochemical capacitor that is accompanied by the charge transfer between electrolyte and electrode. Heteroatoms doping, such as oxygen, nitrogen, boron, sulfur, halogens and phosphorus, is an effective approach to change electron density, provide an electrocatalytic surface in the carbon materials in carbon materials so that improve pseudo-capacitance of carbon materials for supercapacitors (Poh et al., 2013).

The presence of oxygen-containing functional group on carbons is unavoidable, since the dangling free radicals remaining from high temperature treatment are highly reactive (Li W et al., 2020). The basic oxygen functional groups, such as carbonyl, carboxyl, can be formed when the biochar is exposed to atmospheric environment (Kinoshita, 1988; Boehm, 1994). On the other hand, the activation process usually contributes to the addition of acidic oxygen functionalities to the carbon surface (Kinoshita, 1988).

**FIGURE 3:**
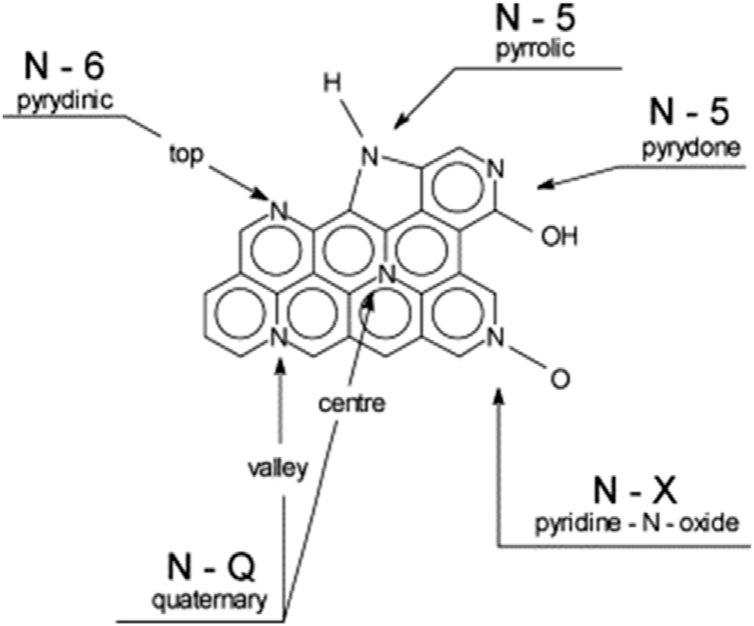
Distribution of nitrogen atoms in carbon graphene structure (Jurewicz et al., 2003)

Ammoxidation is an industrial process that dope elemental N into benzene ring *via* pyrolysis. The N-enrichment activated carbon can be obtained by thermochemical treatment with ammonia or its derivatives, such as urea, ammonium carbonate, hydrazine and hydroxylamine (Bimer et al., 1998; Jurewicz et al., 2002; Jurewicz et al., 2003). The effect of nitrogen on the properties of carbon materials depends on several factors, including size of the graphene planes, number and type of defects, the presence of other heteroatoms and its location in the graphene network, as shown in Figure 3 (Jurewicz et al., 2003). During pyrolysis, pyridinic nitrogen is gradually reformed into quaternary nitrogen from around 450°C; while the pyrrolic and pyridine nitrogen will be converted to pyridinic nitrogen as temperature increased above 800°C (Jurewicz et al., 2002). It is worth noting, however, that the continuous increase in nitrogen content does not always lead to the increase in capacitance because the addition of nitrogen could be at the expense of surface area. The nitrogen groups deposited on the surface of the carbon materials might block pores so that significantly decrease the accessible surface areas (Candelaria et al., 2012).

#### 3.1.3 Lignin-derived carbonaceous composite materials

Despite the fact that transition metal oxide nanoparticles offer superior redox-derived electrochemical pseudocapacitance, metal oxides usually have a high electrical resistance, which leads to a low power density for supercapacitor application (Cheng et al., 2011). A practical strategy is to incorporate transition metal oxide nanoparticles into conductive materials, but the synthesis process requires binder material to integrate all the components. Using a lignin-derived carbon/transition metal oxide composite offers an inexpensive approach to improve electric conductivity of supercapacitor electrode with a binder-free matrix. MnO_2_ nanocrystals were successfully deposited on the surfaces of lignin-derived ECNF mats by hydrothermal degradation of aqueous KMnO_4_ and the MnO_2_/ECNF composite exhibited a capacitance of 171.6 F/g at 5 mV/s (Youe et al., 2018). Iron oxide nanoparticles embedded in lignin-derived hollow carbon nanofiber were synthesized by a coaxial electrospinning process, which delivered a specific capacitance of 121.5 F/g at 5 A/g (Yu et al., 2018). With the assistance of Pluronic F127, a NiO-containing mesoporous carbon material was synthesized through pyrolysis. When applied as a supercapacitor electrode, the prepared MPC/NiO composite exhibited a superior specific capacitance of 880.2 F/g at a current density of 1 A/g (Chen et al., 2013).

### 3.2 Lithium-ion batteries

Batteries, such as LIBs, are the most widely used energy storage technology for electric transportation and portable consumer electronics (Hadjipaschalis et al., 2009). The important criteria to evaluate an electric energy storage material are energy density, power density, efficiency, life span and costs (Musolino et al., 2010). Energy density is measured in watt-hours per kilogram by determining the amount of energy that a device can store in a given volume, while power density is measured in watts per hour by determining the amount of power that a device can generate in a given volume. LIBs have a higher energy density than supercapacitors, while a supercapacitor has a higher power density than a LIB. This difference makes LIBs capable of storing more energy; while supercapacitors release energy much faster. Another important characteristic that must be considered when comparing these two devices are the life span during charging/discharging cycles. Supercapacitors exhibit a cycle life that is two orders of magnitude larger than that of lithium batteries under full charging/discharging (Hadjipaschalis et al., 2009). Therefore, rate performance, as well as cycling performance are among the most importance characteristics to evaluate the electrochemical performance of a lithium-ion battery.

**FIGURE 4:**
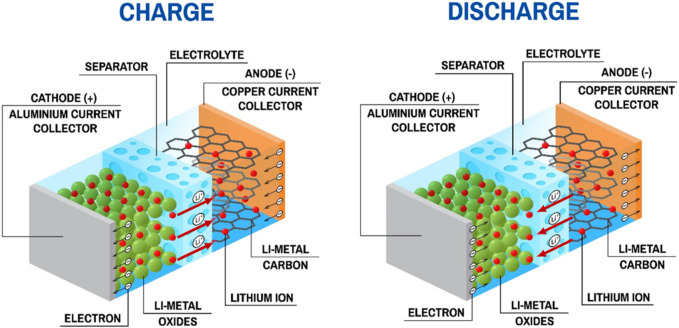
Configuration and working principle of LIBs.

Lithium is the lightest metal as well as the most electropositive element LIBs (Hadjipaschalis et al., 2009). The configuration of a LIB consists of a cathode (Li^+^ host material) and an anode (with Li^+^ accessible inter-atomic sites), which are immersed in electrolyte and isolated by a separator, as shown in Figure 4. The intercalation and deintercalation of lithium-ions causes electrons transfer between cathode and anode during charging and discharging, which fulfill the conversion between chemical energy and electrical energy (Winter and Brodd, 2004).

#### 3.2.1 Lignin-derived non-graphitic carbon LIB anode

Since LIBs became commercially available in 1991, graphitic carbon materials have become the most widely applied negative electrode material for LIBs. Graphite is a crystal of elemental carbon, which is formed by graphene layers bonded by van der Waals forces (Evans, 1908). The intercalation of Li^+^ between the graphene layers allows good electrical conductivity, mechanical stability and Li-ions dispersion, which makes graphite an attractive anode material with low cost but moderate energy density and good cycling stability (Nitta et al., 2015). However, for graphite, at the end of Li intercalation, it is thermodynamically favorable to form LiC_6_, which makes graphite have a theoretical capacity limitation of 372 mAh/g (Jian et al., 2017).

Non-graphitic carbons are generally able to be divided into “hard carbon” and “soft carbon” in terms of bonding strength between sp2 layers (graphene) (Irisarri et al., 2015). Hard carbon can be derived from precursors with high oxygen content, such as lignin and cellulose fractions (Jian et al., 2017). A strong cross linking of hard carbon crystallites immobilizes the carbon layers and incorporates the crystallites in a rigid mass (Franklin and Sciences, 1951). Soft carbon is typically synthesized from aromatic hydrocarbons with low oxygen content, such as petroleum and coal. The layers of soft carbon are mobile under weak cross linking, which is eligible to be converted to graphite-like crystallites under high temperature (Jian et al., 2017).

With the better understanding of mechanism model for ions intercalation and the development of diverse modification strategies for carbonaceous materials, a variety of non-graphitic carbon materials have been evaluated as anode material for LIB application (Dahn et al., 1995). Of all synthetic carbon-based materials, hard carbon materials attracted extensive investigation as LIB anode materials. The “house of cards” model was proposed to explain the intercalation mechanisms of hard carbon in LIB, as well as sodium-ion battery (SIB) negative electrode (Gupta and Harrison, 1994). The small graphitic grains with disordered orientation in hard carbons not only contribute to the insertion sites of Li-ions between graphene layers but also provide nanopores and defects that offers additional gravimetric capacity, allowing a higher capacity than the theoretical capacity limitation of graphite (Flandrois, 1999). Low cost renewable carbon precursors, such as cellulose and lignin, can be easily converted to hard carbon materials, therefore, received increasingly interests as negative electrode materials of LIBs (Stevens and Dahn, 2000; Lu and Zhao, 2017).

The first attempt at lignin-derived hard carbon for LIB anode application can be dated back to 1980s. A series of pyrolytic carbonaceous materials derived from biomass-based precursors, including lignin, were direct mounted with lithium metal and evaluated as anode material of LIB, which offered improved stability and capacity (Hayashi and Satoh, 1986). Afterwards, with the hypothesis that the nanovoids and pore openings of hard carbon contribute to a large capacity for LIB, a variety of hard carbons derived from sugars, endocarps, woods, and lignin under different operation conditions (pyrolysis temperature, heating rate and flow rate of argon) were characterized with XRD, SAXS, and BET, and lignin-derived hard carbon exhibited a capacity of 440 mAh/g as anode material for LIBs (Xing et al., 1996). In order to acquire lignin-derived hard carbon with higher capacity, various synthetic strategies have been applied to develop nanocarbon materials, including carbon fiber, mesoporous carbon and graphene. Blending with 10% of polyethylene oxide, lignin-derived electrospun carbon fiber mat delivered a capacity of 412 mAh/g at a current density of 30 mA/g (Wang S et al., 2013). Mesoporous carbon materials offer extraordinary interface between electrode and electrolyte and facilitate ion transfer and diffusion in the anode. A hierarchical mesoporous carbon material generated from lignin demonstrated a capacity of 470 mAh/g after 400 cycles at a current density of 200 mA/g (Zhang et al., 2015a). The theoretical capacity of graphene is as high as 744 mAh/g, which is much higher than that of graphite. A graphene-like carbon/Fe_3_O_4_ nanocomposite material derived from black liquor was characterized as LIB anode material and exhibited a capacity of 750 mAh/g after 1,400 cycles at a current density of 1A/g (Yi et al., 2017).

Although the theoretical limitation of graphite can be broken through diverse modification or synthetic approaches, the capacity of carbon-based LIBs is still relatively low. Therefore, several alternative active materials have been extensively evaluated to replace graphite as next-generation negative electrodes. Of which, silicon has received significant attention as an alternative to the graphitic carbon.

#### 3.2.2 Lignin-derived carbon/silicon nanoparticle composite as LIB anode

With a specific capacity of about 3,600 mAh/g, silicon has been considered as one of the most promising candidates to replace graphite as active material of next-generation LIB anode (Ma et al., 2014). However, recent studies have suggested that electromechanical degradation due to irreversible volume change, delamination and fracture of Si particles is responsible for the irreversible capacity loss of the silicon-based LIBs. The electromechanical degradation raises two issues. 1) the loss of physical contact between electrode and current collector caused by repeatedly large volume expansion change during insertion and extraction of Li ions. The capacity and electronic and ionic conductivity of electrode degrade quickly without the physical contact. 2) endless development of the solid electrolyte interphase (SEI) on the freshly damaged Si particles surface will drain lithium and electrolyte, which further lead to the capacity fade of LIBs. Therefore, diverse strategies have been developed to improve the electrochemical performance of Si-based lithium-ion batteries (Jin et al., 2017), including structurally engineered Si (e.g. nanostructured Si (Szczech and Jin, 2011), 3D porous Si particles (Kim et al., 2008), coating (Shin and Cho, 2018)), flexible current collector (Choi et al., 2013), pre-lithiation (Forney et al., 2013), electrolyte additives (Chen et al., 2014), and improved binder materials (Bridel et al., 2009; Magasinski et al., 2010; Kovalenko et al., 2011a).

Among these strategies, composite material combining nanostructured Si core with a protecting shell has been considered as a promising strategy (Wang B et al., 2013; Chen et al., 2016a). Taking advantage of this strategy, numerous anode materials with innovative structural design have been developed, e.g., carbon-coated Si NPs (Ng et al., 2006), porous Si nanowires (Kim and Cho, 2008) and Si nanotubes (Park et al., 2009), graphene and its chemical modified derivatives, reduced graphene oxide encapsulated Si NPs (Wang et al., 2010; Wu et al., 2011; Luo et al., 2012; Tang et al., 2015). Similarly, several lignin-derived carbon/silicon core-shell nanoscale composites have been examined as anode of LIBs. Rios et al. embedded Si nanoparticles on electrospun carbon fibers which derived from organosolv hardwood lignin in a core-shell structure. As a result, the composite retained a specific capacity about 400 mAh/g at a current density of 0.84A/g after 40 cycles (Rios et al., 2014). Niu et al. synthesized a self-assembly lignin coated silicon nanoparticles composite under the assistance of phytic acid, which exhibited a 2,950 mAh/g at a current density of 0.3A/g after 100 cycles. Most recently, Chen et al. developed a lignin-derived C/Si NPs composite *via* a one-pot, binder-free pyrolysis process, which showed 1,390 mAh/g at a current density of 0.54A/g after 100 cycles (Chen et al., 2016b). Afterwards, by optimizing pyrolysis temperature from 800°C to 600°C while keep other condition the same, the lignin-derived C/Si NPs composite exhibited an enhanced electrochemical performance by retaining 2,378 mAh/g after 100 cycles at 1 A/g (Chen et al., 2016a). The synthesis process represented a comparable electrochemical performance when Si NPs were replaced with SiO_x_ nanoparticles. After 250 cycles, the lignin-derived C/SiOx composite maintained about 900 mAh/g at 0.2 A/g when tested as a LIB anode (Chen et al., 2017).

### 3.3 The impact of impurities on the electrochemical performance

Unlike carbon precursors from fossil fuels, technical lignins contain a variety of elemental impurities derived from ash content and fractionation processes. For example, kraft lignin may contain elemental impurity of Al, Ca, Cu, Fe, K, Mg, Mn, Mo, Na, P and S in forms of inorganic minerals and organic constituents (Fahmi et al., 2007). The presence of the elemental impurities has been reported adversely affecting the bio-oil yield during fast pyrolysis of biomass. Ash forming elements, even at trace levels (<0.1%), can alter both the thermal degradation rate and chemical pathways during biomass pyrolysis (Evans and Milne, 1987). The alkali metals potassium (K) and sodium (Na), and the alkaline Earth metals calcium (Ca) and magnesium (Mg), are known to catalyze the thermal degradation of biomass to light gases rather than the preferred liquids (Patwardhan, 2010). Other studies have demonstrated negative effects of higher bulk ash content on the pyrolysis process yield (Das et al., 2004; Fahmi et al., 2008).

However, the study associated with the impact of impurities in lignin on the reaction chemistry of the carbonization (slow pyrolysis) process and the inconsistency in physiochemical, structural and electrochemical properties of the resulting carbon materials is rare. It was found that the ash content (mainly Na_2_CO_3_ and Na_2_SO_4_) in the kraft lignin exhibited a high reactivity during CO_2_ activation, which caused rapid collapse or blockage of micropores of resulting activated carbon (Carrott et al., 2008). However, the influence of impurities on the development of porous structure and thus the electrochemical performance remains to be further investigated.

## 4 Recent advances of understanding process-structure-property-performance relationship

The increasing demand for high performance energy materials requires the materials to equip a set of properties. These properties can be classified into thermal, mechanical, physical, chemical. The superior properties are attributed to the unique structures of materials, which are the result of synthesis and processing. Therefore, a better understanding of the process-structure-property-performance relationship is critical to reveal mechanisms of a prepared materials, and in turn to direct the design of energy storage materials with an enhanced performance. Herein, we summary recent advances associated with disclosing the process-structure-property-performance relationship for the lignin-derived energy storage materials.

### 4.1 Thermochemical conversion process: Pyrolysis-GC-MS

Despite thermochemical conversion processes, including slow pyrolysis and hydrothermal carbonization, play a critical role to the synthesis of carbonaceous functional materials, the study that correlates process conditions, such as carbonization temperatures, heating rates, residence time, choice of inert environment, activation temperatures, activation methods (agents) and ratios, etc., with physiochemical properties and electrochemical performance of synthesized materials is rare. Furthermore, contradictory results have been reported in the limited literature regarding how process conditions affect the performance of lignin-derived carbon material for energy storage applications. For example, with black liquor as carbon precursor and KOH as activation agent, Zhang et al. systematically examined the effect of operation conditions, including KOH/lignin ratio (1:1 to 1:5), carbonization temperature (500°C and 800°C) and activation temperature (700, 800°C and 900°C) on electronical performance of supercapacitors. It was found that the electrode carbonized at 500°C and activated at 800°C with KOH/lignin ratio of 4:1 exhibited highest specific capacitance of 286.7 F/g at a current density of 0.2 A/g and retained 207.1 F/g at a high current density of 8 A/g (Zhang et al., 2015b). However, in a separate study, under the same conditions (black liquor as precursor, KOH as activation agent, carbonization temperature: 500°C, activation temperature: 800°C), the reported specific capacitance and SSA were 35 F/g and 2,694.5 m^2^/g, respectively at a low current density of 10 mA/g (Zhao et al., 2010). Take another study as example, Chen et al. reported a lignin-derived C/Si composite electrode through a slow pyrolysis process. When lignin and Si nanoparticles were copyrolyzed at 800°C, the synthesized anode material delivered a specific capacity of 1,390 mAh/g, as compared to a much higher specific capacity of 2,378 mAh/g obtained at a pyrolysis temperature of 600°C. Therefore, in addition to exploring the potentials of numerous novel carbon precursors, investigating novel structure, such as core-shell, hollow, hierarchy, etc., establishing a comparable quantitative analysis method to correlate the process-structure-property-performance based on deep understanding of lignin pyrolysis chemistry process is necessary for lignin-derived carbonaceous materials.

Gas chromatography–mass spectrometry (GC-MS) is an analytical technology that unites the features of gas-chromatography and mass spectrometry to identify and quantify different substances within a single test. By coupling a micro-pyrolyzer with a GC-MS, volatiles evolved from pyrolysis can be separated by GC column and then transfer to the MS and flame ionization detector (FID) to be further identified and quantified. The use of GC-MS as an analytical tool to investigate pyrolysis of lignin is dated back to the late of 1970s, when Kraft lignin was first attempted to be collected in a “captive sample”, only five phenolic compounds (phenol, guaiacol, cresol, 4-methylguaiacol, and 4-ethylguaiacol) were successfully quantified due to the limitations of analytical technology of the time (TJiang et al., 2010). Afterwards, numerous studies have been conducted aiming to obtain a mechanistic insight into lignin depolymerization during pyrolysis. However, all of those studies were focusing on fast pyrolysis, which was favored for its capacity of maximized converting biomass into liquid fuel, namely bio-oil (Bridgewater and Peacocke, 2000; S; Czernik and Bridgewater, 2004; Patwardhan, 2010; TJiang et al., 2010; Li et al., 2017).

**FIGURE 5:**
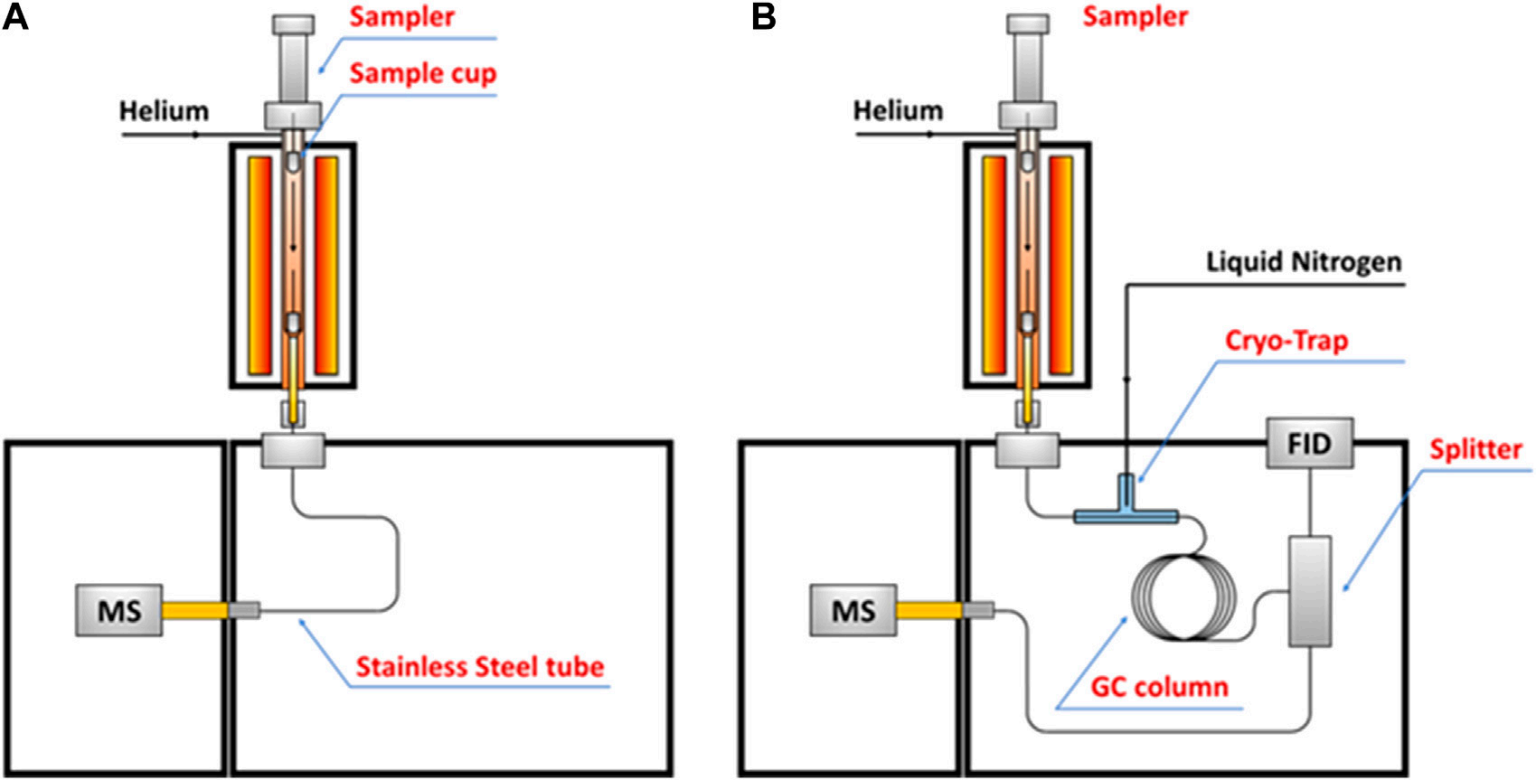
Schematic diagrams of **(A)** EGA-MS and **(B)** HC-GC-MS in this study (Li W et al., 2020).

The increasing demands for low costs while high performance materials attract attention to converting lignin into diverse carbonaceous functional materials. However, unlike fast pyrolysis of lignin, which has been extensively investigated, the process of lignin slow pyrolysis has not been quantitatively examined, primarily because 1) the multi-phased volatiles were hard to be recovered and tracked over longtime course during slow pyrolysis; 2) process conditions with a very low heating rate are hard to be well controlled. Until recently, with assistance of a cryo-trap that installed at the head of GC column so that selectively recover volatiles evolved in a well-controlled condition (temperature regions, heating rates), lignin slow pyrolysis was, for the first time, quantitatively investigated *via* a combination of EGA-MS and HC-GC-MS analyses (Li W et al., 2020). The configuration of pyrolysis-GC-MS for the EGA-MS and HC-GC-MS is shown in Figure 5. This study provides critical insights into the slow pyrolysis chemistry of lignin and the properties of the resulting carbon materials. In a separate study, the process-structure-property-performance relationship of a 3-dimensional, interconnected lignin-derived carbon/silicon (C/Si) composite electrode for lithium-ion battery application was investigated *via* EGA-MS and HC-GC-MS analysis (Li et al., 2021). It was found the elemental Si and C of the C/Si NPs were linked *via* O rather than direct Si-C bond. There is a subtle balance between the Si-O-C bond and more stable and stronger Si=O, which can be tailored by controlling thermal conversion conditions (Li et al., 2021). The balanced bonding overlayer on the surface of Si NPs may serve to alleviate the mechanical degradation through either restricting excessive volume change during electrochemical cycling or forming a shell to protect the Si NP core from redundant lithiation. This study establishes a link between synthesis condition, properties of C/Si nanocomposite materials and their electrochemical performance and durability as electrodes in the next-generation LIBs. Furthermore, the Py-GC-MS analytical method has potential to be extended into studies of other functional material synthesis, including but not limited to lithium-ion battery applications (Li et al., 2021).

### 4.2 Mechanical property: Scratching test

It is believed the extensive volume change of Si nanoparticles leads to the pulverization and delamination of electrode layer, which significantly impairs the mechanical contact between the electrode materials and current collector (Ryu et al., 2004). Therefore, in order to assure electronic conductivity and sufficient mechanical integrity of electrode material, a number of polymeric binders were assessed, such as carboxymethyl cellulose (CMC), Nafion, sodium alginate (SA) and polyvinylidene fluoride (PVDF) (Liang et al., 2004; Li et al., 2007; Kovalenko et al., 2011b). However, the correlations between electromechanical stability and binder properties have not been fully understood. Some researchers believe that a superior electronic conductivity and mechanical integrity are able to be achieved by introducing a highly extensible elastomeric binder which is tolerant of huge volumetric changes (Chen et al., 2003); while others proposed that stiffer binders serve better in maintaining conductivity and mechanical integrity of Si-based composite electrodes than those elastomeric alternatives (Li et al., 2007; Kovalenko et al., 2011b). Nevertheless, a recent study found that both the flexible Nafion and the stiffer SA demonstrated better electrochemical performance than PVDF did when served as binder material of Si composite electrodes (Xu et al., 2017), though both hardness and elastic modulus of PVDF are in between those of Nafion and SA. Additionally, an adhesion property between electrode material and current collector is also believed critical to electrochemical performance of Si-based composite electrodes (Chen et al., 2006). However, PVDF exhibited an much inferior electrochemical performance when used as binder material of Si composite electrodes, despite that PVDF-Cu interface has a much stronger adhesion strength than SA-Cu, (Hu et al., 2018). Therefore, it is believed that neither the mechanical property of binder material, nor the adhesive strength, but the cohesive strength between the binder and active materials affects the electrochemical performance the most (Hu et al., 2018).

**FIGURE 6:**
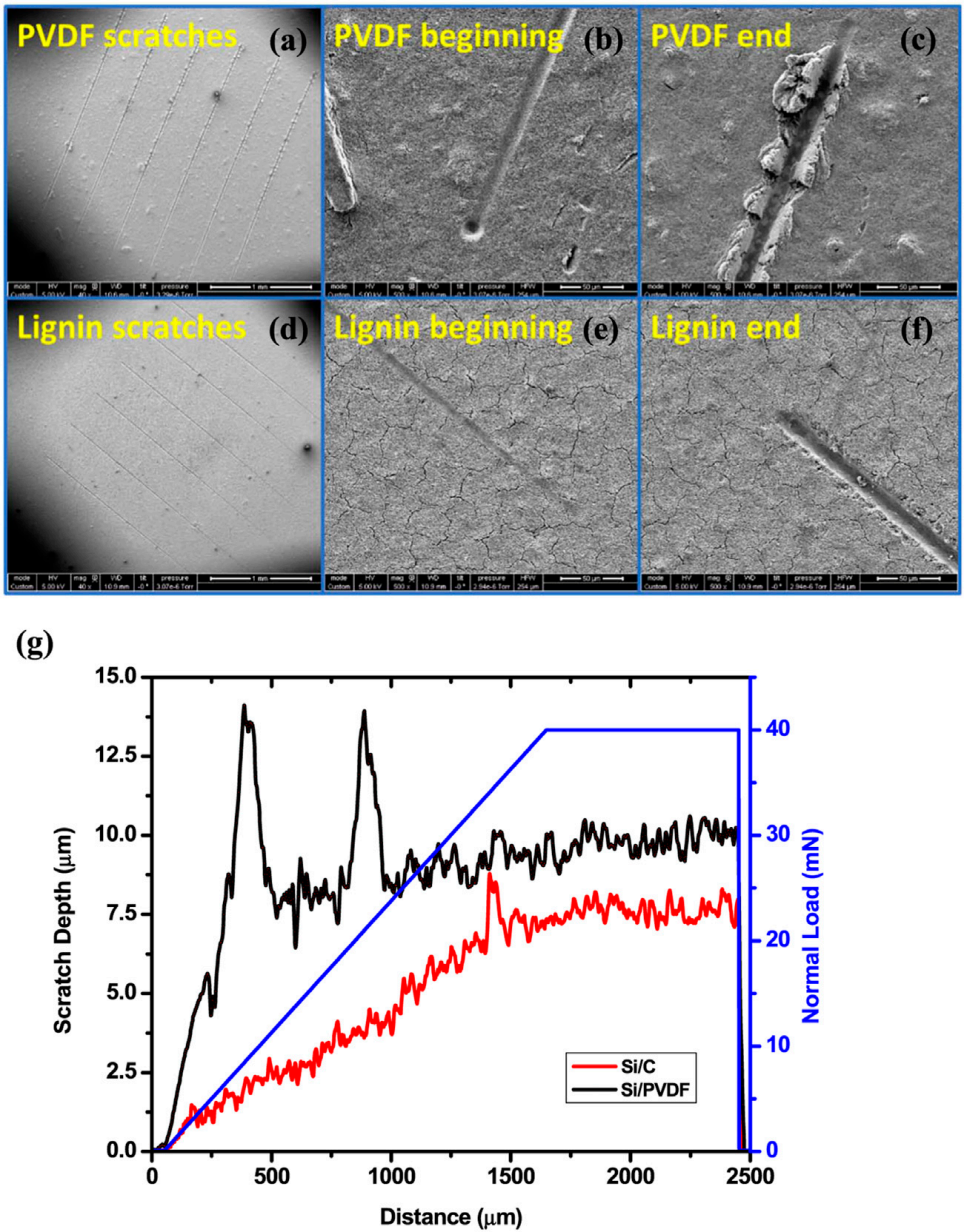
Scratch test results of the PVDF and KL electrodes: SEM images of the **(A)** overall, **(B)** beginning and **(C)** end of micro-scratch tracks for the electrode with PVDF binder and **(D)** overall, **(E)** beginning and **(F)** end of micro-scratch tracks for the electrode with KL binder; **(G)** Scratch depth profiles of the PVDF and KL binders as a function of scratch distance (Li et al., 2021).

In order to assess the cohesive strength of a lignin-derived C/Si composite electrode for lithium-ion battery application, a set of scratch tests were conducted, as shown in Figure 6. The scratch test results demonstrated a superior cohesion strength between Si NPs and C, which can be attributed to the binding interactions formed among Si, C, and O during lignin slow pyrolysis. It is because the unique chemical bonding structure formed at a specific process condition (around 600°C) significantly improved the electrochemical properties of the C/Si composite electrodes.

### 4.3 Mechanical property: Environmental nanoindentation

**FIGURE 7:**
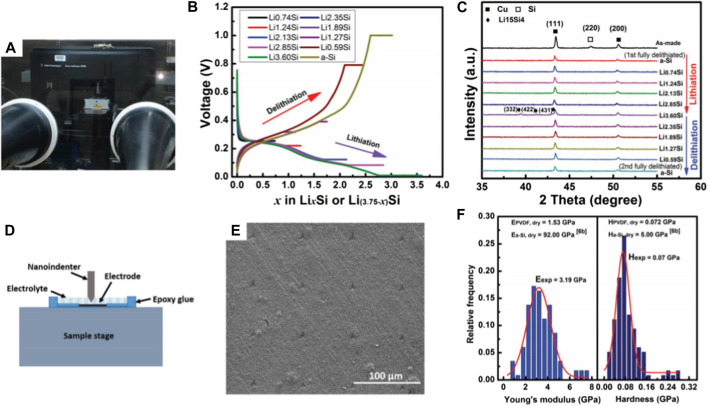
**(A)** The G200 nanoindentation system inside an argon-filled glovebox; **(B)** Voltage profiles of Si electrodes corresponding to different SOCs during the 2nd cycle; To obtain a homogeneous Li distribution, the galvanostatic mode was followed by a potentiostatic mode until the current density dropped below 1.5 μA cm^−2^; **(C)** XRD patterns of composite electrodes with different SOCs; **(D)** The schematic diagram of the liquid cell for nanoindentation under wet condition; **(E)** A typical nanoindentation matrix in the Si composite electrode after the 1st full delithiation; **(F)** The distribution histograms of Young’s modulus and hardness of the Si composite electrode after the 1st full delithiation under dry condition (Wang and Cheng, 2017).

It is challenging to measure mechanical properties of the electrodes after electrochemical tests because, 1) some of the SEI components, such as LiOH and R-CHOLi, are highly reactive when exposed to air and moisture (Malmgren et al., 2013); 2) crystalline intermetallics of Li and Si are thermodynamically unstable, it is easy to be oxidized in air (Gu et al., 2013). Therefore, to perform mechanical measurement, as well as sample preparation in an inert environment offers a solution to overcome those challenges. By installing a nanoindentation system inside an argon-filled glovebox, mechanical properties of Si composite electrodes before and after electrochemical tests under dry and wet condition (liquid electrolyte) were determined (Wang et al., 2018). With the same environmental nanoindentation method, the viscoplastic properties of pure Li metal were also successfully characterized (Wang and Cheng, 2017), as shown in the Figure 7. As an effort to establish a correlation among thermochemical process, mechanical property and electrochemical performance of lignin-derived C/Si composite electrode for lithium-ion application, mechanical properties (elastic modulus and hardness) of electrodes synthesized at 600°C and 800°C (pyrolyzed at 2°C/min in argon environment) were measured (Chen et al., 2016a). The composite electrodes synthesized at 600°C demonstrated a significantly stiffer property in comparison to those at 800°C. The environmental nanoindentation method has potential to be extended to a wide variety of energy storage materials.

### 4.4 *In-situ* measurement of the changes in structure, composition, and morphology of composite electrodes

Recently, extensive efforts have been made to obtain real time observations and measurements toward the evolution of lithiation and de-lithiation processes on atomic and molecular scale structure, morphology, chemical composition and mechanical properties and acquire a deeper mechanistic insight on SEM and stress development during electrochemical cycling. By using an *in-situ* X-ray transmission microscopy (TXM) (Chao et al., 2010) and an *in-situ* transmission electron microscope (TEM) (Liu and Huang, 2011) and *in-situ* atomic force microscopy (AFM), the evolution of the interior microstructure and morphology changes of working composite anodes for Li-ion battery have been successfully observed. *In-situ* NMR were developed to examine the electrochemical intercalation of lithium ions into three different anodes, including graphite, corannulene, and sepiolite clay-derived carbon (Gerald Ii et al., 2000), Si (Key et al., 2009). Lithiation-induced mechanical properties play a critical role to the performance and durability of lithium-ion batteries. *In-situ* mechanical properties and stress evolution of thin film graphite electrode during electrochemical cycling were measured (Mukhopadhyay et al., 2011). Recently, a mathematical model was proposed, based on the measurement of curvature evolution in respect with modulus and stress of Si composite electrode during lithiation and de-lithiation (Li D et al., 2020).

### 4.5 Molecular dynamics simulation

**FIGURE 8:**
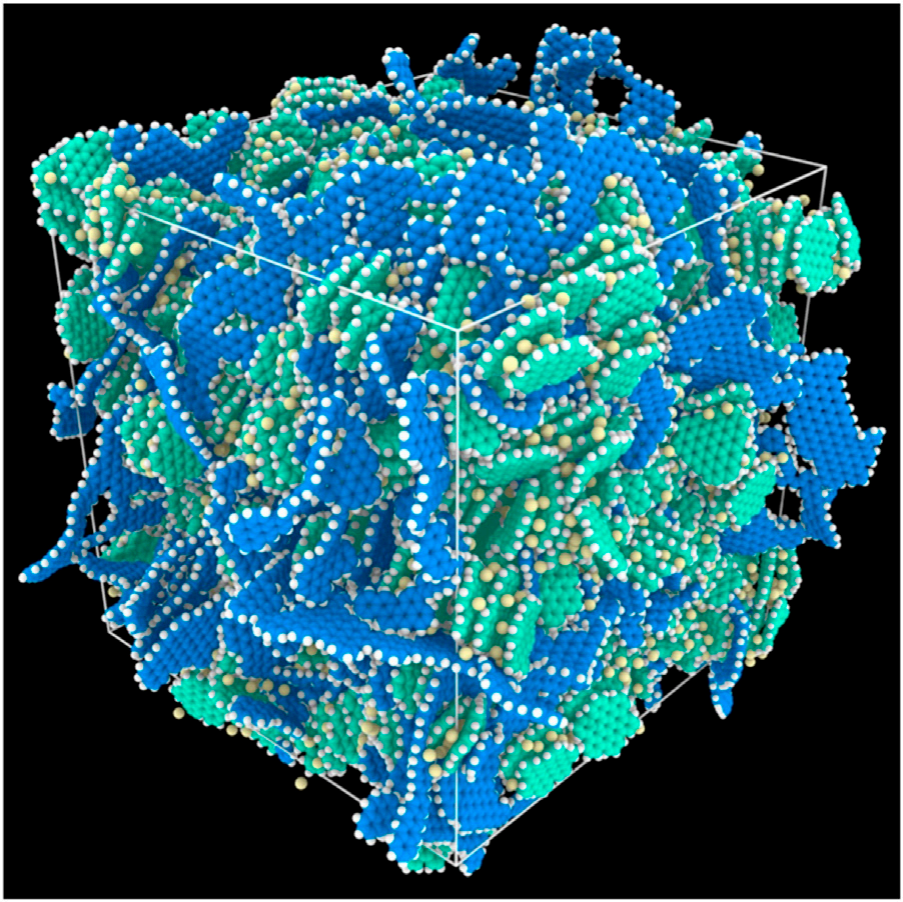
The hierarchical carbon anode model after equilibration. Atoms are colored by type: Amorphous carbon is blue, crystalline carbon is green, hydrogen atoms are white, and lithium ions are yellow. This simulation corresponds to a loading of 147.8 mAh g^−1^ and an initial condition in which all Li atoms were initially intercalated within the crystalline domain (McNutt et al., 2017b).

Molecular dynamics simulation was used to develop atomistic models associated with interactions among the amorphous and crystalline domains of lignin-derived non-graphitic carbon LIB anodes. Lignin-derived non-graphitic carbon LIB anodes are composed of amorphous and nanocrystalline domains. The nanocrystalline carbon with size of 5–17 Å in radius are dispersed within the amorphous carbon matrix. Molecular dynamics simulation mechanistically explains an experimental observation that spacing between planes of nanocrystalline carbon is inversely correlated with crystallite size and suggests that the structural configuration of crystallites is a function of entropy (McNutt et al., 2014b). Through molecular dynamics simulation and neutron diffraction, the relationship structural properties, including crystallite size, intracrystalline *d* spacing, crystalline volume fraction and composite density, was built up by pair distribution functions (McNutt et al., 2014a). Based on molecular dynamics simulation, a novel mechanism about lithiation of lignin-derived hierarchical carbon LIB anode was proposed. It is believed that the Li ions are actually intercalated at the interface of crystalline carbon domains rather than between layers of carbon as they do in graphite carbon LIBs anode, as shown in Figure 8, because the edges of the crystalline and amorphous carbon of the lignin-derived non-graphitic carbon composites are terminated by hydrogen atoms, which play a crucial role in adsorption (McNutt et al., 2017b). As an attempt to establish correlation among process, structure, property and performance of carbon composite LIBs, in a separate study, energetics of Li-ion binding is examined through associating the changes in energy and charge distribution with variation in structural properties. It was found that the carbon composite with more volume of crystallites has higher Li ions storage capability due to higher interfacial area between the amorphous and crystalline domains. With more volume of crystallites and stable H atoms at the edge of interfaces, therefore, graphite carbon can offers more reactive sites to absorb Li ions (McNutt et al., 2017a).

## 5 Conclusion and perspectives

Currently, the commercial potential of cellulosic biofuels is hindered by lack of appropriate application for the lignin fraction. With extensive research focus in the last decade, great strides have been made in developing lignin valorization strategies. With the rapid development of modern industries and fast-changing electronic technology, the demand for energy storage materials is increasing. Having high carbon content, highly branched and cross-linked structure and low feedstock cost, lignin has been considered as a promising precursor for energy storage materials. However, despite the increasing interest on lignin derived energy storage materials, high cost on lignin fractionation in biorefineries and lack of fundamental understand the mechanism of thermochemical conversion to carbonaceous materials and its effect on structure, properties and performance of the lignin-derived carbonaceous functional materials are hindering its practical applications. Therefore, the following aspects should be considered in the future to develop lignin-derived energy storage materials:

Novel biomass pretreatment/lignin fractionation technologies need to be further developed to achieve high lignin recovery rate at low cost, and more importantly, structural and compositional features need to be tailored for the final products. Efficient lignin fractionation technologies with low costs offer more opportunities for lignin-derived energy storage materials. Although DES pretreatment was demonstrated to be an effective method to fractionate biomass for biorefineries to produce both biofuels and high-quality lignin streams, the costs are still too high. Therefore, reducing costs of DES pretreatment are critical for industrial application. Such efforts include finding low-cost and biocompatible precursors, pretreatment at higher biomass loadings, process integration and intensification. Furthermore, the properties of the DES extract lignin need to be tuned for electrochemical energy storage applications.

Slow pyrolysis has demonstrated to be one of the most commonly used thermochemical conversion methods to convert biomass into various carbon materials. However, the process of biomass slow pyrolysis is so far not well understood because the difficulties of controlling the operation conditions and recovering and tracking the multi-phased volatile products over a long course (Li W et al., 2020). The effect of lignin pyrolysis on the resulting carbon material has been investigated in several studies. However, the connections between operation condition of activation process and lignin-derived AC as well as the possible interactions between carbonization and activation are not clear. Additionally, the variation of pyrolytic biochar and hydrothermal biochar to the functional material application has not been largely explored. Therefore, future work is needed to link the synthesis condition, properties and performance of functional materials.

Since the quality and property of the lignin-derived carbon materials largely rely on the feedstock characteristics and preprocessing conditions, the impurities in the lignin feedstocks have become increasingly major concern. Despite the beneficial effects of doping specific atoms on tailoring the functionality of carbon materials, the impurities in general are more likely to be evenly distributed in lignin-derived carbon matrix rather than doping on the surface of carbon materials. Driven by totally different catalytic systems and reaction mechanisms, the impact of the impurities on the reaction chemistry of the carbonization (slow pyrolysis) process and the inconsistency in physiochemical, structural and electrochemical properties of the resulting carbon materials need to be determined and characterized.

Finally, although it is believed that valorizing lignin into energy storage materials may enhance the economic viability and the success of biorefineries, the extent of coproducing lignin-derived energy storage materials to offset the cost of biofuel production in biorefineries has not yet been recognized. It is a worthwhile effort evaluating feedstock logistics and economic potential of the developed lignin valorization strategy to aid the process design and optimization through coproducing lignin-derived carbon materials along with biofuels in a biorefinery. The results will allow an assessment of the technical maturity and cost of the individual steps and overall process.
